# Combined epithelial-mesenchymal transition with cancer stem cell-like marker as predictors of recurrence after radical resection for gastric cancer

**DOI:** 10.1186/1477-7819-12-368

**Published:** 2014-12-02

**Authors:** Gui-fang Xu, Wei-jie Zhang, Qi Sun, Xinyun Xu, Xiaoping Zou, Wenxian Guan

**Affiliations:** Department of Gastroenterology, Affiliated Drum Tower Hospital of Nanjing University Medical School, 321 Zhongshan Road, Nanjing, 210008 China; Department of General Surgery, Drum Tower Clinical College of Nanjing Medical University, 321 Zhongshan Road, Nanjing, 210008 China; Department of General Surgery, Affiliated Drum Tower Hospital of Nanjing University Medical School, 321 Zhongshan Road, Nanjing, 210008 China; Department of Pathology, Affiliated Drum Tower Hospital of Nanjing University Medical School, 321 Zhongshan Road, Nanjing, 210008 China

## Abstract

**Background:**

The aim of the study was to identify the incidence and the predictors of recurrence after curative resection and the clinical significance of epithelial-mesenchymal transition (EMT) and stem cell-like phenotypes in gastric cancer.

**Methods:**

In a total of 1,463 patients that underwent curative resection for gastric cancer between January 2001 and January 2008 at Drum Tower Hospital, 402 (27.5%) experienced recurrence. They were divided into early recurrence (within two years) and late recurrence (more than two years). The clinicopathological characteristics, including five EMT-related proteins (Snail-1, ZEB-1, E-cadherin, vimentin, and β-catenin) and the gastric cancer stem cell markers CD44 and CD54, therapeutic modalities, survival time after recurrence, and recurrence patterns were compared between the two groups.

**Results:**

Loss of E-cadherin expression and aberrant expression of vimentin and the known gastric cancer stem cell maker CD44 were significantly associated with aggressive clinicopathologic features. Multivariate analysis showed that stage III gastric cancer patients with early recurrence had larger tumors and more lymph node metastasis, coupled with aberrant expression EMT and cancer stem cell marker, than patients with late recurrence. Early recurrence was associated with more distant metastasis than late recurrence and patients tended to die within two years of recurrence.

**Conclusions:**

Combined EMT with cancer stem cell-like marker is a predictor of recurrence after radical resection for gastric cancer. Advanced TNM stage was associated with early cancer death after recurrence.

## Background

Gastric cancer is the fourth most common cancer in the world, and surgery remains the main treatment, with curative intent. Even after gastrectomy and lymphadenectomy, every year many patients die of recurrence. The epithelial-mesenchymal transition (EMT), a developmental process in which epithelial cells show reduced intercellular adhesion and acquire migratory fibroblastic properties, is considered to be critical for invasive and metastatic progression in cancer [1]. The process of EMT is associated with the downregulation of epithelial markers, abnormal translocation of β-catenin, and aberrant upregulation of mesenchymal markers. Cancer stem cells are cells within a tumor that possess self-renewal and tumor-initiating capacities. In gastric cancer, CD44-positive tumor cells were shown to have cancer stem cell properties, including the tumor-initiating ability [2], and Chen *et al*. [3] showed that CD44 + CD54+ cells exhibit cancer stem cell capabilities in gastric cancer tissues. These tumor-initiating cells provide a reservoir that can cause tumor recurrence after therapy [4].

Previously, Ryu *et al*. [5] showed that a combination of EMT and stem cell-like phenotypes is an important predictor of aggressive biologic behavior of gastric cancer; however, few studies currently have been investigating EMT with cancer stem cell-like marker in gastric cancer with recurrence and prognosis. The aim of the current study was to characterize recurrence patterns, identify the predictors of time of recurrence and initial recurrence patterns of gastric cancer with early and late recurrence, and find risk factors to predicate early recurrence after radical resection for gastric cancer. This could help in the preoperative assessment of appropriate therapeutic strategies and in providing more intensive and tailored follow-up strategies for high-risk patients.

## Methods

### Patient selection

Between January 2001 and January 2008, excluding patients who were lost to follow-up or poor compliance, a total of 1,463 patients underwent curative resection for gastric cancer at Drum Tower Hospital. A total of 402 (27.5%) of these patients experienced recurrence and were subsequently included in the study; thus the recurrence rate in our study was 27.5%. The mean age of the 402 patients was 64.7 years (range: 26 to 91 years), with a male:female ratio of 1.87:1. Medical charts and pathology reports were reviewed to record clinical and pathological data. To evaluate whether the expression profiles of the various EMT markers were different between primary and metastatic gastric cancers, cases with regional lymph node metastasis were also evaluated. In terms of the timing of recurrence, some tumors recur within the first year after resection, and in these cases, inadequate radical surgical approach or systematic metastasis at operation may be suspected. In our study, we defined early recurrence as recurrence within two years after surgery. Late recurrence was defined as recurrence more than two years after surgery. These 402 patients were divided into two groups, namely the early recurrence group, which contained 248 patients, and the late recurrence group, which contained 154 patients. Exclusion criteria included synchronous gastric double cancer, a previous history of surgery for gastric cancer, gastric stump cancer, or cancer anatomically classified as esophageal cancer according to the American Joint Committee on Cancer (AJCC) seventh edition [6].

The study was approved by the clinical research ethics committees of the Drum Tower Hospital and informed consent was obtained from all patients.

### Tissue array and immunohistochemistry

All samples in the study were removed from formalin-fixed paraffin-embedded gastric cancer tissues. For all of the arrays, three cores from different areas of the tumor in each case were removed and placed into a new blank recipient paraffin block as previously described by Hsu *et al*. [7], and 4-μm thick sections were obtained for immunohistochemistry. The specimens were deparaffinized and dehydrated by a graded series of ethanol solutions(Nanjing Chemical Reagent Co., Ltd., Nanjing, China). The immunohistochemical staining for EMT-related proteins Snail-1, zinc finger E-box-binding homeobox 1 (ZEB-1), vimentin, E-cadherin, β-catenin, and cancer stem cell-like markers CD44 and CD54, were performed and evaluated according to the previous method [3, 5].

As for statistical analysis of multiple markers, the specimen was recorded as having positive immunoreactivity for each antibody, excluding E-cadherin, if more than 5% of the cancer cells were immunoreactive, and tumor cells with less than 5% were considered as negative. The scoring criteria for E-cadherin were defined as ‘positive (intact)’ when immunoreactivity on the cell membrane was present in more than 25% of the gastric cancer cells.

### Patterns of recurrence

Patterns of recurrence reported represent the first sites of documented recurring disease after curative resection. Recurrence was documented from the first clinical or radiological signs of disease with an unrelenting course leading to tumor progression and/or death. Confirmation through biopsy was recommended for any evidence of recurrent disease or distant metastases. Recurrence patterns were classified as locoregional, peritoneal, or hematogenous. Locoregional recurrence was defined as any cancer recurrence at the resection margin or lymph nodes (including regional nodes as well as retropancreatic and para-aortic nodes) or operation bed within the region of the resection (below the diaphragm and liver, and above the pancreas and abdominal wound). Peritoneal recurrence was defined as any cancer recurrence within the abdominal cavity due to intraperitoneal distribution including visceral metastasis, rectal shelf, pericholedochal, and periureteral infiltration. Hematogenous recurrence was defined as any metastatic lesion detected in liver, lung, bone, and other distant organs.

### Follow-up

After surgery, all patients were followed up on regularly. Follow-up assessments were performed every three months for the first five years after surgery, and every six months thereafter until the patient’s death. The follow-up procedures included a medical history, physical examination, routine blood tests, liver function tests, measurement of tumor marker levels (carcinoembryonic antigen and carbohydrate antigen 199), a chest radiograph, and other imaging studies. All routine procedures were performed by a surgeon, esophagogastroduodenoscopy was performed by a gastroenterologist, and upper gastrointestinal series, abdominal sonogram, and computed tomography (CT) scans were performed by a radiologist. For confirmed recurrent disease or distant metastases, biopsies were not obtained for new, multiple pulmonary lesions or for lesions characteristic of osseous metastases noted on CT or whole-body bone scans. When metastasis was suspected, further techniques were utilized, such as bone scan, positron emission tomography (PET), and biopsy sampling.

### Statistical analysis

All values are expressed as the mean ± standard deviation. Categorical variables were analyzed using the χ^2^ test and continuous variables using the independent-samples t-test. Logistic regression multivariate analysis was conducted to determine the differences between the two groups. Analysis of survival was performed by the Kaplan-Meier method, and differences between the curves were tested using a two-tailed log-rank test. *P* <0.05 was considered to be statistically significant. Statistical analysis was performed using SPSS (SPSS 13.0 for Windows; SPSS Inc., Chicago, Illinois, United States) software.

## Results

### Expression of epithelial-mesenchymal transition and cancer stem cell-like marker association with tumor recurrence

Table 1 shows the clinicopathologic characteristics of the patients with early recurrence and late recurrence. Among the perioperative variables recorded for univariate analysis were: presence of CD44, CD54, and vimentin, loss of E-cadherin and β-catenin expression, with a tumor size of 5 cm or more, more advanced T-stage, and a more advanced TNM stage with early recurrence to late recurrence Multivariate analysis was employed to identify the independent risk factors for overall recurrence. The analysis showed that patients presenting with a tumor size of 5 cm or more, and a more advanced TNM stage, coupled with EMT expression and expression of cancer stem cell marker CD44, were prominent in early recurrence (Table 2).

**Table 1:** **Clinicopathological characteristics of recurrent gastric cancer patients**

	Early recurrence (n = 248) n (%)	Late recurrence (n = 154) n (%)	***P***value
Age, years			0.392
<65	109	61	
≥65	139	93	
Gender, male/female	162/86	100/54	0.937
Tumor size, cm			0.013
<5	76	68	
≥5	172	86	
Gastrectomy			0.061
Total	91	71	
Subtotal	157	83	
Location			0.232
Upper	46	27	
Middle	49	20	
Lower	130	95	
Diffuse	23	12	
Gross appearance			0.001
Superficial tumor	18	25	
Bormann type I and II	25	40	
Bormann type III and IV	205	89	
Histology type			0.315
Differentiated	76	40	
Undifferentiated	172	114	
Lauren’s classification			0.572
Intestinal type	104	69	
Diffuse type	144	85	
Lymph node metastasis			0.002
Negative	52	54	
Positive	196	100	
Lymphovascular invasion			0.003
Absent	57	56	
Present	191	96	
Depth of invasion			<0.001
T1 and T2	46	53	
T3 and T4	202	101	
TNM stage			<0.001
I	14	27	
II	43	59	
III	191	68	
Lymph node dissection			0.414
D1	109	75	
D2	86	54	
D3	53	25	
EMT expression (%)			
Aberrant expression of mesenchymal marker			
Snail-1	117(47.2)	64(41.6)	0.271
ZEB-1	168(67.7)	109(70.8)	0.522
Vimentin	87(35.1)	21(13.6)	<0.001
Expression loss of epithelial marker			
E-cadherin	112(45.2)	35 (22.7)	<0.001
β-catenin	29 (11.7)	19(12.3)	0.846
Expression of cancer stem cell marker (%)			
CD44	163(65.7)	71(46.1)	0.001
CD54	120(48.3)	63(40.9)	0.037
Adjuvant chemotherapy, yes/no	221/27	142/12	0.308

**Table 2:** **Multivariate analysis of factors independently associated with the timing of recurrence (early versus late)**

	Early	Late	***P***value	Odds ratio	95.0% CI for experiment B
Tumor size ≥5 cm	172(69.4)	86(55.8)	0.001	8.750	(2.265 - 33.799)
TNM stage III	191(77.0)	68(44.2)	0.040	0.493	(0.251 - 0.970)
EMT expression (%)					
Vimentin	87(35.1)	21(13.6)	0.031	2.359	(1.082 - 5.145)
E-cadherin	112(45.2)	35(22.7)	<0.001	0.125	(0.046 - 0.338)
Expression of cancer stem cell marker (%)					
CD44	163(65.7)	71(46.1)	0.017	0.297	(0.108 - 0.806)

### Patterns of recurrence

The median time to recurrence was 17.0 months (range: 2.0 to 89.0). Most recurrences in our study group (248 out of 402, 61.7%) occurred within two years after curative surgery (Table 3). In the early recurrence group, the most common pattern was hematogenous metastasis, followed by locoregional and peritoneal recurrence. Including patterns that developed concurrently, locoregional and hematogenous recurrence was the most common pattern, followed by peritoneal recurrence. In the late recurrence group, the most common pattern was locoregional and peritoneal recurrence, followed by hematogenous recurrence as single patterns, and when patterns that developed simultaneously were included, locoregional recurrence was still the most common pattern. Distant metastasis is more common in early recurrence, but peritoneal dissemination is not significant. Among patients with hematogenous metastasis, liver metastasis is more common in early recurrence.

**Table 3:** **The pattern of initial recurrence of gastric cancer**

	Early recurrence (n = 248) n (%)	Late recurrence (n = 154) n (%)	***P***value
Locoregional recurrence	161(64.9)	85(55.2)	0.052
Hepatoduodenal ligament	57(23.0)	37(24)	0.810
Perigastric area	25(10.1)	24(15.6)	0.101
Anastomosis	23(9.3)	15(9.7)	0.877
Remnant stomach	15(6)	12(7.8)	0.601
Peripancreatic area	12(4.8)	9(5.8)	0.660
Abdominal wall	10(4)	7(4.5)	0.804
Lymph node	51(20.6)	27(17.5)	0.455
Mixed	10(4)	3(1.9)	0.251
Distant metastasis	207(83.5)	109(70.8)	0.003
Peritoneal dissemination	106(42.7)	62(40.3)	0.624
Hematogenous metastasis	112(45.2)	49(31.8)	0.008
Liver	64(25.8)	23(14.9)	0.010
Lung	25(10.1)	17(11)	0.760
Bone	9(3.6)	4(2.6)	0.570
Adrenal gland	7(2.8)	3(1.9)	0.584
Brain	2(0.8)	0	0.264
Skin	2(0.8)	0	0.264
Mixed	3(1.2)	2(1.3)	0.938

### Survival time after recurrence

**Figure 1:**
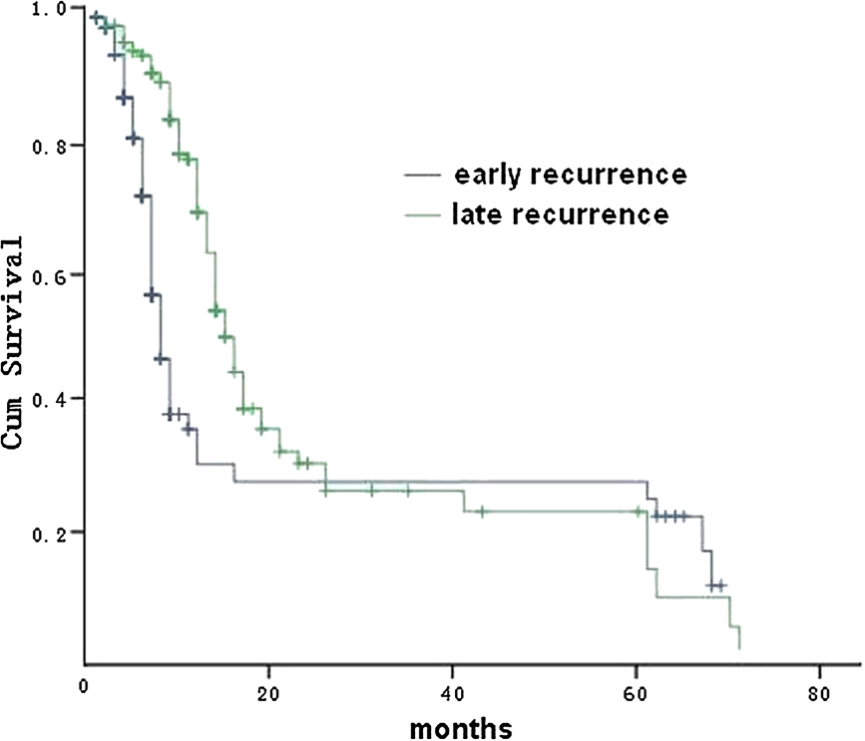
**Comparison of survival after recurrence by Kaplan-Meir.**

The majority of patients with recurrence of gastric cancer (321 out of 402, 79.9%) died within two years after recurrence. The median survival time after recurrence was 8.1 months in early recurrence and 13.3 months in late recurrence, respectively. A total of 16 patients survived more than five years after recurrence: 10 with early recurrence and six with late recurrence. Among the 10 patients with early recurrence, the recurrence pattern included one liver metastasis, two lung metastasis, three anastomosis recurrences, two hepatoduodenal ligament recurrences, one abdominal wall recurrence, and one peritoneal seeding. Among the six patients with late recurrence, the recurrence patterns included one abdominal incision recurrence, two liver metastases, one remnant stomach relapse, one hepatoduodenal ligament recurrence, and one anastomosis recurrence with liver invasion. Survival time after recurrence was significantly shorter in the early group (*P* <0.001, log-rank test) (Figure 1).

## Discussion

Gastric cancer represents the fourth leading cause of cancer mortality worldwide. Locoregional relapse may occur after curative resection, including adjacent lymph nodes resection and multidisciplinary treatment, possibly in the form of peritoneal carcinosis and/or distant metastases, which is one reason for the high rate of recurrence. Carcinoma relapse is essentially lethal, and so far there are no specific treatments to avoid recurrence [8–10].

The clinical significance of EMT has been reported in various human cancers, and specific mechanisms involved in cancer progression, such as the evasion of apoptosis, resistance to chemotherapy, and the acquisition of stem cell-like properties and their influence on patient survival have been suggested [1, 5, 11, 12]. CD44 is a transmembrane glycoprotein that is well known as a cancer stem cell marker in gastric cancer [2]. CD44 is positively and significantly associated with tumor metastasis, recurrence, and mortality in gastric cancer [13, 14].

In the present study, expression of mesenchymal marker vimentin was more frequent in early recurrence than in late recurrence cancers (*P* <0.001), and E-cadherin expression was lost in 112 (45.2%) of 248 early recurrence cancers, but only 35 late recurrence (22.7%) cancers showed loss of E-cadherin expression (*P* <0.001). These findings demonstrate that the transformation of the gastric epithelial cells could promote tumor recurrence and metastasis. CD44 and CD54 expressions were also found more frequently in early recurrence patients than those of late recurrence (*P* = 0.001, *P* = 0.037), suggesting that stem cell markers could be taken as a promoter for tumor progress.

There have been several studies [1, 5] on the complexity of the interactions between EMT-related proteins and their effects on cancer recurrence. The present study determined that the combination of alterations in the protein expression of vimentin, E-cadherin, and CD44 was the most effective prognostic factor in gastric cancer. In addition, this combination was an independent predictive factor of recurrence, together with a conventional clinicopathologic parameter such as pTNM stage and histologic differentiation.

In our series, there is a large difference in cancer size, gross appearance, lymph node metastasis, lymphovascular invasion, depth of invasion, and stage between the early and late recurrence groups, and patients with CD44 and vimentin, and loss of E-cadherin and β-catenin expression, have a higher tendency towards early recurrence. Our results showed that patients presenting with a tumor size of 5 cm or more, and a more advanced TNM stage, coupled with EMT expression and expression of cancer stem cell marker CD44, were prominent in early recurrence in the multivariate analysis.

As for recurrence patterns, in general, there are three main recurrence patterns of gastric cancer after curative surgery: locoregional recurrence, peritoneal dissemination, and hematogenous metastasis. Our data showed that patients with early recurrence had more distant metastasis, but not peritoneal dissemination, than those with late recurrence. Hematogenous metastasis was more common in early recurrence, which was similar to some studies [9, 10]. There is no significant difference between early and late recurrence with respect to locoregional recurrence. We found that liver, lung, and bone are the most frequently hematogenous metastatic organs. This result concurs with others [10, 15]. Liver metastases show a tendency to occur earlier, whereas lung and bone metastases occur later. The liver is the first filter of cancer cells through the portal venous blood flow of the stomach; the lung functions as a secondary filter. This result suggests that patients should be monitored carefully for hematogenous metastasis during the first two years of follow-up, and for locoregional or peritoneal recurrence subsequently.

Survival times after recurrence have rarely been documented in previous studies, although a new study performed by Wu *et al*. [16] reported that most patients succumbed within one year of receiving a diagnosis of recurrence, with the median survival time after recurrence being only 6.7 months. In our results, the median survival time of the early recurrence group was significantly lower (8.06 versus 14.97 months, *P* <0.001). Despite poor survival after recurrence of cancer and an unfavorable response to the chemotherapy in some patients, 16 patients had long-term survival of more than five years after recurrence. We suggest that close follow-up with B-ultrasound and endoscopies are important, especially in the first two years, in order to prolong survival.

## Conclusions

Hematogenous metastasis was found to be common in patients that experienced recurrence within two years of curative resection, and patients with large tumor size (≥5 cm) and advanced TNM stage (stage III) were found to be more prone to early recurrence. EMT and stem cell-like phenotypes are correlated with aggressive clinical features in gastric cancer, and the three proteins, E-cadherin, vimentin, and CD44, may be the best combination for predicting patient recurrence.

## Abbreviations

EMT: Epithelial-mesenchymal transition
ZEB-1: Zinc finger E-box-binding protein 1
CD44: Cluster of differentiation 44
CD54: Cluster of differentiation 54
AJCC: American Joint Committee on Cancer
CT: Computed tomography
PET: Positron emission tomography
SPSS: Statistical product and service solutions
TNM: ‘Tumor, node, metastases’.
